# Critical role of triglycerides for adiponectin levels in hepatitis C: a joint study of human and HCV core transgenic mice

**DOI:** 10.1186/s12865-021-00445-5

**Published:** 2021-08-11

**Authors:** Ming-Ling Chang, Jing-Hong Hu, Li-Heng Pao, Ming-Shyan Lin, Chia-Jung Kuo, Shiang-Chi Chen, Chun-Ming Fan, Ming-Yu Chang, Rong-Nan Chien

**Affiliations:** 1 Division of Hepatology, Department of Gastroenterology and Hepatology, Chang Gung Memorial Hospital, No 5, Fu Hsing Street, Kuei Shan, Taoyuan, Taiwan; 2 Department of Medicine, College of Medicine, Chang Gung University, Taoyuan, Taiwan; 3 Department of Internal Medicine, Chang Gung Memorial Hospital, Yunlin, Taiwan; 4 Graduate Institute of Health-Industry Technology, Chang Gung University of Science and Technology, Taoyuan, Taiwan; 5 Research Center for Industry of Human Ecology, Chang Gung University of Science and Technology, Taoyuan, Taiwan; 6 Department of Cardiology, Heart Failure Center, Chang Gung Memorial Hospital, Yunlin, Taiwan; 7 Department of Nursing, Taipei Medical University, Taipei, Taiwan; 8 Department of Biomedical Sciences, Chang Gung University, Taoyuan, Taiwan; 9 Division of Pediatric Neurologic Medicine, Chang Gung Children’s Hospital, Taoyuan, Taiwan; 10 Division of Pediatrics, Chang Gung Memorial Hospital, Keelung, Taiwan; 11 Liver Research Unit, Department of Gastroenterology and Hepatology, Chang Gung Memorial Hospital, No 5, Fu Hsing Street, Kuei Shan, Taoyuan, Taiwan

**Keywords:** HCV, Adiponectin, Triglycerides, HOMA-IR, SVR, DAA, HCV core

## Abstract

**Background:**

Both hepatitis C virus (HCV) infection and adiponectin are critically involved in metabolism. The reversal and associations of altering adiponectin levels after sustained virological responses (SVRs) following direct-acting antivirals (DAA) in HCV-infected patients remained elusive.

**Methods:**

A joint study was conducted in a prospective cohort of 427 HCV-infected patients and a line of HCV core transgenic mice.

**Results:**

Of 427, 358 had completed a course of DAA therapy and 353 had SVRs. At baseline, male sex (95% CI β: − 1.44 to − 0.417), estimated glomerular filtration rate (eGFR) (− 0.025 to − 0.008), triglycerides (− 0.015 to − 0.005), and fibrosis-4 levels (0.08–0.297) were associated with adiponectin levels; BMI (0.029–0.327) and triglycerides levels (0.01–0.03) were associated with homeostatic model assessment for insulin resistance (HOMA-IR) in HCV-infected patients. At 24-week post-therapy, in SVR patients, male sex (− 1.89 to − 0.5) and eGFR (− 0.02 to − 0.001) levels were associated with adiponectin levels, levels of BMI (0.094–0.335) and alanine transaminase (0.018–0.078) were associated with HOMA-IR; compared with baseline levels, adiponectin levels decreased (6.53 ± 2.77 vs. 5.45 ± 2.56 μg/mL, *p* < 0.001). In 12-month-old HCV core transgenic mice with hepatic steatosis, triglyceride levels (0.021–0.111) were associated with adiponectin levels, and hepatic adipopnectin expression was comparable with that of control mice.

**Conclusions:**

Triglycerides and hepatic fibrosis are associated with HCV-specific alteration of adiponectin levels, and adiponectin may affect insulin sensitivity through triglycerides during HCV infection. In DAA-treated patients, after SVR, adiponectin levels decreased and the linking function of triglycerides between adiponectin and insulin sensitivity vanished. Moreover, HCV core with hepatic steatosis might affect extrahepatic adiponectin expression through triglycerides.

**Supplementary Information:**

The online version contains supplementary material available at 10.1186/s12865-021-00445-5.

## Background

Hepatitis C virus (HCV) is a human pathogen responsible for acute and chronic liver disease, chronically infecting an estimated 71.1 million individuals worldwide [1], and classified into 8 genotypes [2]. In addition to hepatic complications including steatosis, liver cirrhosis and hepatocellular carcinoma (HCC), HCV infection induces several extrahepatic complications, such as hypolipidemia, insulin resistance (IR), diabetes and cardiovascular events [3, 4]. Thus, HCV is now considered to cause metabolic alterations instead of being simply a viral infection. The reversal of HCV-associated metabolic alterations after sustained virological response (SVR) following interferon-based studies had been well demonstrated [3, 5–10]. However, some HCV-associated cardiometabolic events cannot be reversed, and HCV-associated HCC is also not eradicable after SVR [3, 11]. Moreover, the reversal of metabolic alterations might be biased by interferon-based therapy, as interferon therapy has been associated with increases in lipid levels [12] and with immune modulation in the patients [13]. With the advent of direct-acting antivirals (DAAs), which target specific proteins of HCV during its life cycle [14], anti-HCV treatment has resulted in a high cure rate with a short treatment duration in patients with chronic HCV infection (CHC), and the reversal of HCV-associated metabolic alterations might not be biased by any interferon-related effects.

Adiponectin, a 30-kDa adipokine, is mainly expressed in adipocytes [15], while increased visceral adipose tissue stores reduce serum adiponectin [16]. It is the most abundantly secreted adipokine [17]. Several IR-associated hormones such as insulin and catecholamines dysregulate adiponectin expression [18]. Post-translational adiponectin modifications result in the secretion of oligomers of 90-kDa trimers, found in the circulation. Adiponectin might protect hepatocytes from triglyceride accumulation by increasing β-oxidation, decreasing fatty acid de novo synthesis, and promoting the uptake and inhibiting the production of glucose in the liver [19, 20]. Hepatic steatosis and hyperlipidemia are therefore usually associated with low adiponectin levels [20, 21]. Moreover, adiponectin possesses anti-inflammatory, anti-atherosclerotic and anti-apoptotic properties [22]. Paradoxically, circulating adiponectin levels have been associated with cardiovascular events and all-cause mortality [23–25]. Because both HCV infection and adiponectin are deeply involved in metabolism, their relationship might aid to probe the therapeutic targets for HCV-associated cardiometabolic complications. In CHC patients with SVRs following interferon-based therapy, adiponectin may affect insulin sensitivity through triglycerides. After viral clearance, adiponectin levels were directly associated with insulin sensitivity and decreased upon improved hepatic fibrosis [8]. However, how adiponectin levels evolve after SVR in CHC patients following DAA therapy remained unidentified and is crucial for the patients' prognosis, since interferon-based therapy had been replaced by DAA therapy as the standard of care treatment for CHC currently [26]. Being a HCV capsid protein, HCV core possesses the ability to interact with a variety of cellular components [27] and is the least variable of the 10 HCV proteins in variant viruses emerging constantly in patients [28]. Our previous studies based on HCV core transgenic mice had shown that hepatic HCV core expression affects lipid metabolism genes [29], elicits mitochondrial stress [30], exhibits the topological and evolutional relationships with hepatic lipid vesicles [31] and alters the serum adiponectin levels in the non-obese mice with hepatic steatosis [32]. By taking advantage of this HCV core transgenic mice, the associated basis for HCV-associated adiponectin level alteration might be uncovered.

Accordingly, we sought to elucidate the impact of HCV infection on adiponectin levels in a prospective study of CHC patients underwent DAA therapy. In parallel, the associated basis was elucidated via the serum biochemistry and immunohistochemistry (IHC) studies of the HCV core transgenic mice [29–32].

## Results

### Baseline characteristics of CHC patients

Of 427 CHC patients, 232 (54.3%) were female patients, the mean age was 61.3 years. The males had higher levels of body mass index (BMI), alanine aminotransferase (ALT), the homeostatic model assessment for insulin resistance (HOMA-IR), uric acid (UA), model for end-stage liver disease (MELD) score and ferritin and lower levels of total cholesterol (TC) and adiponectin than the females (Table 1).

**Table 1:** Baseline characteristics of the CHC patients

	All (n = 427)	Male (n = 195)	Female (n = 232)	*p* values *
Age (years)	61.3 ± 12.8	59.1 ± 12.1	61.3 ± 12.8	0.076
BMI (kg/m^2^)	24.6 ± 3.9	25.1 ± 3.46	24.2 ± 4.19	0.012
*HCV genotype*				
Genotype 1, n (%)	256 (59.9)	116 (59.4)	140 (60.3)	0.31
Genotype 2, n (%)	137 (32.0)	60 (30.7)	67 (28.8)	0.95
Genotype 3, n (%)	6 (1.7)	4 (2)	2 (0.8)	0.301
Log HCV RNA (logIU/mL)	5.98 ± 0.87	5.96 ± 0.92	5.98 ± 0.83	0.813
ALT (U/L)	79.8 ± 90.4	93.4 ± 108.4	68.5 ± 71.6	0.006
eGFR (mL/min/1.73 m^2^)	86.9 ± 41.7	89.1 ± 40.5	85.0 ± 42.8	0.326
TG (mg/dL)	102.6 ± 52.6	105.9 ± 58.5	99.8 ± 47.1	0.235
TC (mg/dL)	169.5 ± 35.3	163.7 ± 33.0	174.4 ± 36.5	0.002
Fasting glucose (mg/dL)	97.79 ± 31.41	100.10 ± 39.07	96.04 ± 23.96	0.066
Insulin (μIU/mL)	10.18 ± 30.01	10.53 ± 15.50	9.92 ± 37.29	0.793
HOMA-IR	3.17 ± 4.88	3.72 ± 6.61	2.77 ± 2.56	0.045
Uric acid (mg/dL)	5.71 ± 1.62	6.17 ± 1.69	5.28 ± 1.42	< 0.001
NLR	1.98 ± 1.24	2.06 ± 1.19	1.91 ± 1.29	0.248
Platelet (10^3^/uL)	176.8 ± 71.7	176.8 ± 68.2	176.7 ± 74.8	0.996
Liver cirrhosis, n (%)	92 (21.5)	40 (20.9)	52 (22.4)	0.699
Fibrosis-4 score	3.53 ± 3.39	3.22 ± 3.23	3.79 ± 3.51	0.083
MELD score	9.08 ± 3.08	9.51 ± 4.00	8.69 ± 3.62	0.022
Ferritin (ng/mL)	376.1 ± 513.2	461.7 ± 448.9	298.1 ± 555.3	0.002
Adiponectin (μg/mL)	6.52 ± 2.71	5.87 ± 2.37	7.06 ± 2.85	< 0.001
IFNL3-rs12979860 CC genotype, n (%)	1018 (84.7)	166 (85.1)	195 (84.0)	0.829

CHC: chronic hepatitis C virus infection; DAA: direct-acting antivirals; BMI: body mass index; HCV: hepatitis C virus; RNA: ribonucleic acid; ALT: alanine transaminase; eGFR: estimated glomerular filtration rate; TG: triglycerides; TC: total cholesterol; HOMA-IR: homeostatic model assessment for insulin resistance; NLR: neutrophil lymphocyte ratio; IFNL3; interferon-λ3. *, *p* values between male and female CHC patients

### Baseline associations in CHC patients

At baseline, for all 427 CHC patients, male sex, estimated glomerular filtration rates (eGFRs) and levels of triglycerides (TG) were negatively, and levels of fibrosis-4 (FIB-4) were positively associated with adiponectin levels (Table 2); levels of BMI and TG and HCV genotype other than 1, 2 and 3 (non-HCV G123) were positively associated with HOMA-IR (Table 3). While we stratified the patients with baseline insulin resistance (IR), non-HCV G123 were positively associated with HOMA-IR among those with baseline IR (Additional file 1: Table S2); BMI levels were associated with HOMA-IR levels among those without baseline IR (Additional file 1: Table S3).

**Table 2:** Associations of adiponectin levels in CHC patients at baseline

Baseline factors	Univariate analyses		Multivariate analyses	
	95% CI of β (β)	*p* values	95% CI of β (β)	*p* values
Male, yes	− 1.74 to − 0.647 (− 1.194)	< 0.001	− 1.44 to − 0.417 (− 0.93)	< 0.001
Age (years)	0.026–0.068 (0.047)	< 0.001	− 0.039 to 0.015 (− 0.012)	0.394
BMI (kg/m^2^)	− 0.194 to − 0.159 (− 0.127)	< 0.001	− 0.098 to 0.052 (− 0.023)	0.545
HCV genotype	− 0.146 to 0.143 (0.014)	0.868		
Log HCV RNA (logIU/mL)	− 0.507 to 0.176 (− 0.165)	0.342		
ALT (U/L)	− 0.002 to 0.005 (0.001)	0.376		
eGFR (mL/min/1.73 m^2^)	− 0.025 to − 0.012 (− 0.019)	< 0.001	− 0.025 to − 0.008 (− 0.017)	< 0.001
TG (mg/dL)	− 0.018 to − 0.008 (− 0.013)	< 0.001	− 0.015 to − 0.005 (− 0.01)	< 0.001
TC (mg/dL)	− 0.004 to 0.013 (− 0.006)	0.183		
HOMA-IR	− 0.177 to − 0.005 (− 0.061)	0.034	− 0.052 to 0.053 (0.00)	0.99
Uric acid (mg/dL)	− 0.312 to 0.077 (− 0.117)	0.237		
NLR	− 0.321 to 0.203 (− 0.059)	0.659		
Platelet (10^3^/uL)	− 0.013 to − 0.006 (− 0.009)	< 0.001	− 0.006 to 0.004 (− 0.001)	0.617
Liver cirrhosis, yes	− 0.391 to 0.948 (0.279)	0.414		
Fibrosis-4 score	0.17–0.324 (0.247)	< 0.001	0.08–0.297 (0.188)	0.001
Ferritin (ng/mL)	− 0.001 to 0.000 (0.000)	0.212		
IFNL3-rs12979860 CC genotype, yes	− 0.471 to 0.421 (0.4752)	0.324		

CHC: chronic hepatitis C virus infection; CI: confidence interval; BMI: body mass index; HCV: hepatitis C virus; RNA: ribonucleic acid; ALT: alanine transaminase; eGFR: estimated glomerular filtration rate; TG: triglycerides; TC: total cholesterol; HOMA-IR: homeostatic model assessment for insulin resistance; NLR: neutrophil lymphocyte ratio; IFNL3; interferon-λ3

**Table 3:** Associations of HOMA-IR levels in CHC patients at baseline

Baseline factors	Univariate analyses		Multivariate analyses	
	95% CI of β (β)	*p* values	95% CI of β (β)	*p* values
Male, yes	0.081–1.964 (1.022)	0.033	− 1.44 to − 0.417 (− 0.93)	0.100
Age (years)	− 0.069 to 0.004 (− 0.032)	0.085	− 0.039 to 0.015 (− 0.012)	0.678
BMI (kg/m^2^)	0.142–0.378 (0.26)	< 0.001	0.029–0.327 (0.178)	0.02
HCV genotype	1.262–5.513 (3.387)	0.002	0.743–5.279 (3.011)	0.009
Log HCV RNA (logIU/mL)	0.001 to 1.072 (0.536)	0.05	− 0.343 to 0.868 (0.248)	0.433
ALT (U/L)	− 0.136 to 0.162 (0.013)	0.865		
eGFR (mL/min/1.73 m^2^)	0.000–0.023 (0.012)	0.043	− 0.013 to 0.023 (0.005)	0.575
TG (mg/dL)	0.015–0.033 (0.024)	< 0.001	0.01–0.03 (0.02)	< 0.001
TC (mg/dL)	− 0.015 to 0.012 (− 0.002)	0.812		
Adiponectin (μg/mL)	− 0.4 to − 0.016 (− 0.208)	0.034	− 0.197 to 0.215 (0.009)	0.932
Uric acid (mg/dL)	− 0.141 to 0.541 (0.2)	0.25		
NLR	− 0.275 to 0.573 (0.149)	0.491		
Platelet (10^3^/uL)	− 0.004 to 0.009 (0.002)	0.489		
Liver cirrhosis, yes	− 0.692 to 1.595 (0.451)	0.438		
Fibrosis-4 score	− 0.183 to 0.188 (− 0.048)	0.49		
Ferritin (ng/mL)	0.000–0.002 (0.001)	0.027	0.000 to 0.002 (0.001)	0.134
IFNL3-rs12979860 CC genotype, yes	− 1.695 to 1.849 (0.077)	0.932		

HOMA-IR: homeostatic model assessment for insulin resistance; CHC: chronic hepatitis C virus infection; CI: confidence interval; BMI: body mass index; HCV: hepatitis C virus; RNA: ribonucleic acid; ALT: alanine transaminase; eGFR: estimated glomerular filtration rate; TG: triglycerides; TC: total cholesterol; NLR: neutrophil lymphocyte ratio; IFNL3; interferon-λ3

### Post-therapy associations in SVR patients

**Fig. 1:**
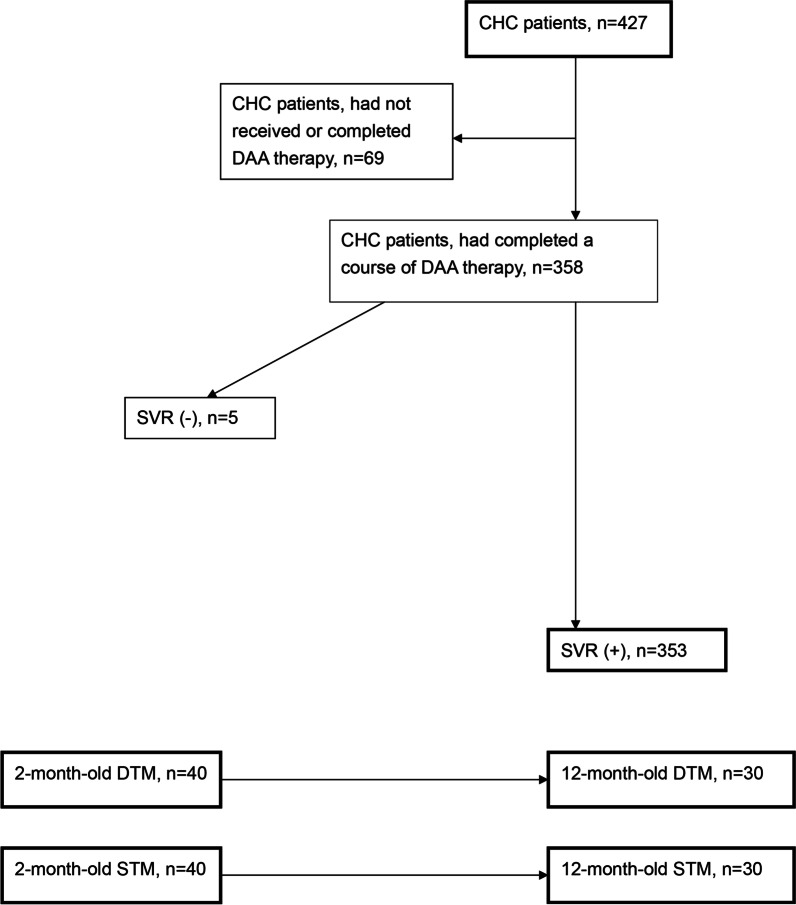
A schematic flow chart of the enrolled patients and transgenic mice. CHC: chronic hepatitis C virus infection; DAA: direct acting antiviral; SVR: sustained virological response; DTM: tTA-HCV core double transgenic mice; STM: tTA single transgenic mice

At 24 weeks post-therapy, among 353 SVR patients (Fig. 1), male sex and eGFRs were negatively associated with adiponectin levels (Table 4); levels of BMI and ALT were positively associated with HOMA-IR (Table 5). While we stratified the patients with baseline IR, among the SVR patients with baseline IR, levels of BMI were associated with HOMA-IR levels (Additional file 1: Table S4); levels of BMI, ALT and neutrophil lymphocyte ratio (NLR) were associated with HOMA-IR levels among SVR patients without baseline IR (Additional file 1: Table S5).

**Table 4:** Associations of adiponectin levels in SVR patients at 24 weeks post-therapy

24-week post-therapy factors	Univariate analyses		Multivariate analyses	
	95% CI of β (β)	*p* values	95% CI of β (β)	*p* values
Male, yes	− 1.708 to − 0.548(− 1.128)	< 0.001	− 1.89 to − 0.5 (− 1.195)	0.001
Age, (years)	− 0.01 to 0.037 (0.013)	0.252		
BMI (kg/m^2^)	− 0.183 to 0.136 (− 0.11)	0.004	− 0.139 to 0.16 (− 0.039)	0.443
ALT (U/L)	− 0.042 to − 0.003 (− 0.023)	0.024	− 0.04 to 0.004 (− 0.018)	0.112
eGFR (mL/min/1.73 m^2^)	− 0.024 to − 0.005 (− 0.015)	0.002	− 0.02 to − 0.001(− 0.011)	0.034
TG (mg/dL)	− 0.008 to − 0.001 (− 0.005)	0.007	− 0.005 to 0.002 (− 0.002)	0.385
TC (mg/dL)	− 0.005 to 0.011 (0.003)	0.418		
HOMA-IR	− 0.153 to 0.045 (− 0.054)	0.283		
Uric acid (mg/dL)	− 0.326 to 0.047 (− 0.14)	0.141		
NLR	− 0.216 to 0.334 (0.059)	0.673		
Platelet (10^3^/uL)	− 0.01 to 0.00 (− 0.005)	0.039		
Liver cirrhosis, yes	− 0.91 to 0.484 (− 0.213)	0.548		
Fibrosis-4 score	0.026 to 0.265 (0.146)	0.017	− 0.044 to 0.197 (21.72)	0.077
Ferritin (ng/mL)	− 0.001 to 0.001 (0.000)	0.904		
IFNL3-rs12979860 CC genotype, yes	− 0.502 to 1.038 (0.268)	0.494		

SVR: sustained virological response; BMI: body mass index; HCV: hepatitis C virus; RNA: ribonucleic acid; ALT: alanine transaminase; eGFR: estimated glomerular filtration rate; TG: triglycerides; TC: total cholesterol; HOMA-IR: homeostatic model assessment for insulin resistance; NLR: neutrophil lymphocyte ratio; IFNL3; interferon-λ3

**Table 5:** Associations of HOMA-IR levels in SVR patients at 24 weeks post-therapy

24-week post-therapy factors	Univariate analyses		Multivariate analyses	
	95% CI of β (β)	*p* values	95% CI of β (β)	*p* values
Male, yes	− 0.2028 to 1.232 (0.515)	0.158		
Age, (years)	− 0.041 to 0.015 (− 0.013)	0.354		
BMI (kg/m^2^)	0.184–0.358 (0.271)	< 0.001	0.094–0.335 (0.215)	0.001
ALT (U/L)	0.049–0.094 (0.071)	< 0.001	0.018–0.078 (0.048)	0.002
eGFR (mL/min/1.73 m^2^)	− 0.0024 to 0.025 (0.011)	0.102		
TG (mg/dL)	0.006–0.014 (0.01)	< 0.001	0.000–0.01 (0.005)	0.057
TC (mg/dL)	− 0.015 to 0.004 (− 0.005)	0.271		
Adiponectin (μg/mL)	− 0.22 to 0.064 (− 0.078)	0.283		
Uric acid (mg/dL)	− 0.1046 to 0.466 (0.181)	0.212		
NLR	− 6.116 to 4.889 (− 0.613)	0.391		
Platelet (10^3^/uL)	− 0.013 to 0.00 (− 0.007)	0.059	− 0.013 to 0.002 (− 0.005)	0.145
Liver cirrhosis, yes	0.12–1.789 (0.954)	0.025	− 1.000 to 1.253 (0.126)	0.825
Fibrosis-4 score	− 0.069 to 0.263 (0.097)	0.252		
Ferritin (ng/mL)	0.001–0.004 (0.002)	0.001	0.000–0.003 (0.001)	0.092
IFNL3-rs12979860 CC genotype, yes	− 1.875 to 0.318 (− 0.778)	0.163		

HOMA-IR: homeostatic model assessment for insulin resistance; SVR: sustained virological response; BMI: body mass index; HCV: hepatitis C virus; RNA: ribonucleic acid; ALT: alanine transaminase; eGFR: estimated glomerular filtration rate; TG: triglycerides; TC: total cholesterol; NLR: neutrophil lymphocyte ratio; IFNL3; interferon-λ3

### Variable alterations in SVR patients

Compared with baseline levels, the SVR patients had decreased adiponectin levels, regardless of sex. Levels of ALT and eGFRs also decreased, while levels of BMI increased. By contrast, no differences were noted between pre- and post-therapy HOMA-IR levels among SVR patients. However, while we stratified the SVR patients by baseline IR, those with and without baseline IR had decreased and increased HOMA-IR levels, respectively (Table 6).

**Table 6:** Comparisons between pre-therapy and 24-week post-therapy variables levels among CHC patients with SVRs

	Therapy	Pre-therapy	24-week post-therapy	*p* values (pre- vs. post-)
**Adiponectin (μg/mL)**				
	Total SVR patients	6.53 ± 2.77	5.45 ± 2.56	< 0.001
	Male SVR patients	5.85 ± 2.35	4.85 ± 2.42	< 0.001
	Female SVR patients	7.15 ± 2.98	5.98 ± 2.58	< 0.001
**HOMA-IR**				
	Total SVR patients	3.25 ± 5.37	2.97 ± 3.09	0.417
	Male SVR patients	3.92 ± 7.39	3.25 ± 3.70	0.491
	Female SVR patients	2.62 ± 2.03	2.71 ± 2.37	0.327
	Baseline IR (+	1.63 ± 0.51	1.99 ± 1.49	0.001
	Baseline IR (−)	5.40 ± 7.69	4.29 ± 4.04	0.01
**BMI**				
	Total SVR patients	24.7 ± 3.9	24.9 ± 3.0	0.002
	Male SVR patients	25.2 ± 3.4	25.5 ± 3.5	0.005
	Female SVR patients	24.5 ± 4.38	24.4 ± 4.51	0.155
**ALT**				
	Total SVR patients	85.0 ± 94.8	23.5 ± 16.0	< 0.001
	Male SVR patients	100.7 ± 121.4	24.8 ± 15.0	< 0.001
	Female SVR patients	71.9 ± 60.4	21.0 ± 14.6	< 0.001
**eGFR**				
	Total SVR patients	92.4 ± 38.5	87.4 ± 36.4	< 0.001
	Male SVR patients	94.8 ± 38.9	88.8 ± 35.8	< 0.001
	Female SVR patients	90.2 ± 38.7	85.8 ± 37.6	0.045

CHC: chronic hepatitis C virus infection; SVR: sustained virological response; HOMA-IR: homeostatic model assessment for insulin resistance; BMI: body mass index; ALT: alanine transaminase; eGFR: estimated glomerular filtration rate

**Fig. 2:**
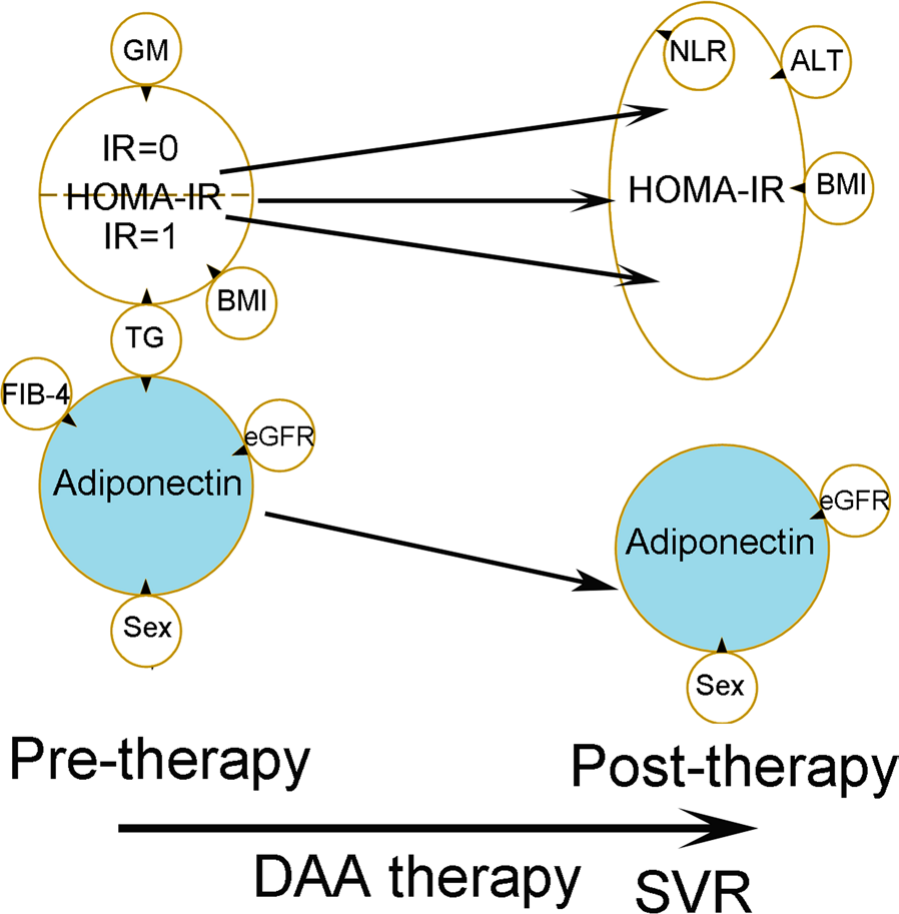
The cross-sectional adiponectin and homeostasis model assessment-estimated insulin resistance (HOMA-IR)-centered associations between dependent and independent factors before (pre-therapy) and 24 weeks after DAA therapy (post-therapy). Tips of black arrowheads: dependent factors; bases of black arrowheads: independent factors; GM: HCV genotype; TG: triglycerides; IR: insulin resistance; BMI: body mass index; FIB-4: Fibrosis-4 index; pre-therapy: levels of variables before direct acting antiviral (DAA) therapy; eGFR: estimated glomerular filtration rate; NLR: neutrophil to lymphocyte ratio; ALT: alanine aminotransferase; SVR: sustained virological response. Rising arrow indicates post-therapeutic increases in HOMA-IR (baseline IR = 0, ie. no baseline IR) levels, while descending arrows indicate post-therapeutic decreases in HOMA-IR (baseline IR = 1, ie. with baseline IR) and adiponectin levels

A summary of the associations and alterations of adiponectin and HOMA-IR levels was shown in Fig. 2.

### Serum biochemistry of the mice

In 2-month-old tetracycline transactivator (tTA)-HCV core double transgenic FVB/N mice (DTM), sex was associated with adiponectin levels; in 12-month-old DTM, sex and TG levels were independently associated with adiponectin levels (Table 7).

**Table 7:** Associations of adiponectin levels in HCV core transgenic mice

	2-month-old DTM				12-month-old DTM			
	95% CI of β (β)	*p* values	95% CI of β (β)	*p* values	95% CI of β (β)	*p* values	95% CI of β (β)	*p* values
Male, yes	− 2.83 to − 1.07 (− 1.90)	< 0.001	− 6.954 to − 0.547 (− 3.75)	0.025	− 7.8 to − 2.5 (− 5.2)	0.001	− 5.616 to − 0.50 (− 3.059)	0.023
ALT (U/L)	0.000–0.007 (0.004)	0.041	− 2.37 to 0.039 (− 0.099)	0.144	− 0.035 to 0.025 (− 0.005)	0.29		
TG (mg/dL)	− 0.056 to 0.048 (− 0.004)	0.866			0.054–0.14 (0.097)	< 0.001	0.021–0.111 (0.066)	0.007
TC (mg/dL)	− 0.136 to 0.007 (− 0.065)	0.073	− 0.172 to 0.107 (− 0.032)	0.622	− 0.147 to 0.043 (− 0.052)	0.257		
HOMA-IR	− 0.343 to 0.221 (− 0.061)	0.662			− 0.891 to 0.493 (− 0.199)	0.536		

HCV: hepatitis C virus; DTM: tTA-HCV core double transgenic mice; ALT: alanine transaminase; eGFR: estimated glomerular filtration rate; TG: triglycerides; TC: total cholesterol; HOMA-IR: homeostatic model assessment for insulin resistance

### IHC studies of the mice

**Fig. 3:**
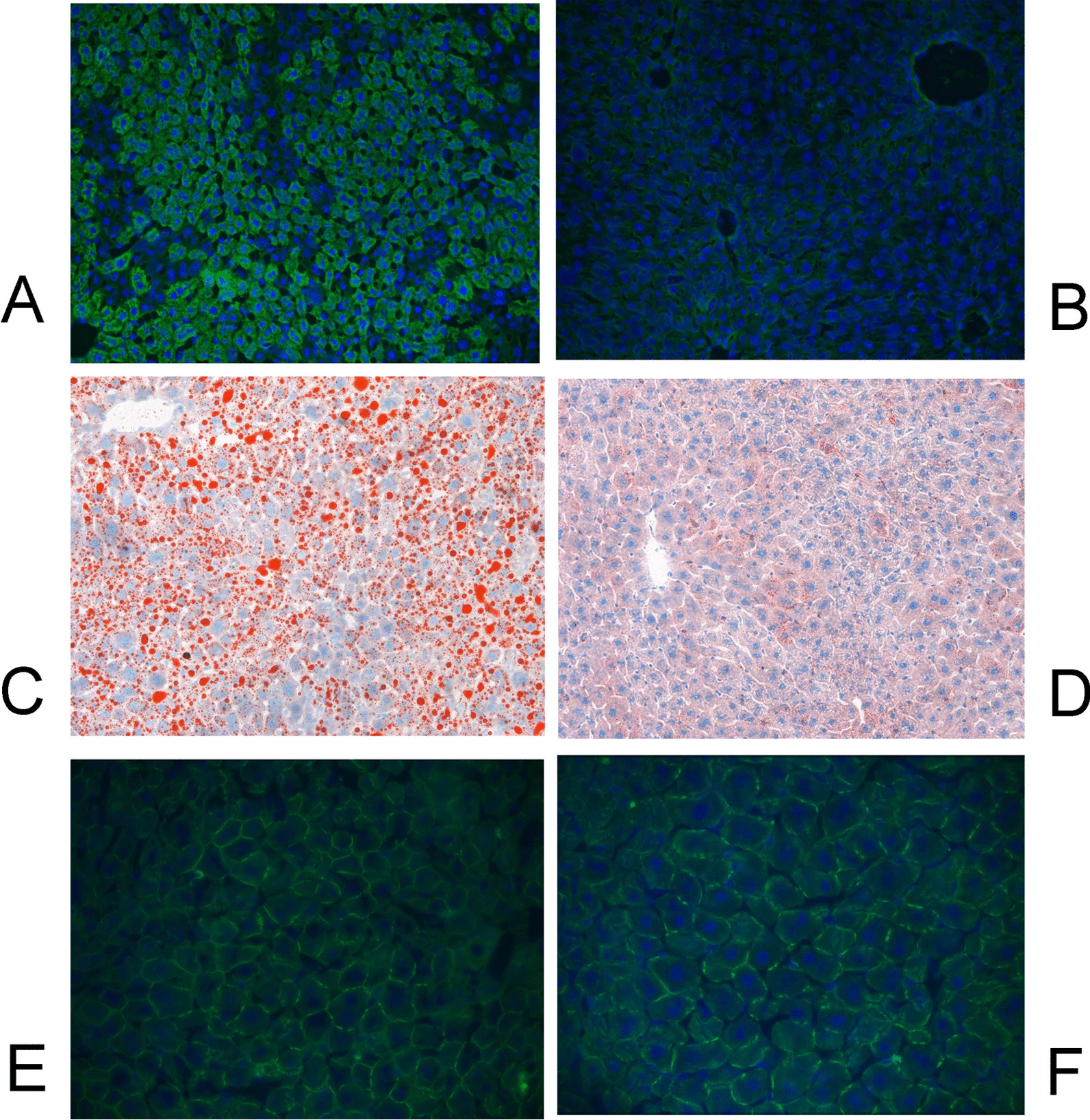
IHC studies for HCV core (**A** and **B**), lipid (**C** and **D**) and adiponectin (**E** and **F**) in the frozen liver samples of tTA-HCV core DTM (**A**, **C** and **E**) and tTA STM (**B**, **D** and **F**). The HCV core-positive cells were shown in green, the intracellular lipids were shown in red, the adiponectin was shown in green (in the cell borders)

The livers of tTA-HCV core DTM showed extensive HCV core expressions (Fig. 3A) and intracellular lipid accumulations (Fig. 3C). By contrast, the livers of tTA DTM did not show any HCV core expression (Fig. 3B) and only exhibited negligible intrahepatic lipids (Fig. 3D). Comparable hepatic adiponectin expressions were noted between the livers of single transgenic mice (STM) and DTM (Fig. 3E, F) (*p* = 0.578).

## Discussion

The most compelling results of the current study are as follows:

(1) At baseline, male sex, eGFRs, levels of TG, and FIB-4 were associated with adiponectin levels; levels of BMI and TG and HCV genotype were associated with HOMA-IR levels in CHC patients. (2) At 24 weeks post-therapy, male sex and eGFRs were associated with adiponectin levels; levels of BMI and ALT were associated with HOMA-IR levels in SVR patients; compared with baseline levels, adiponectin levels decreased, HOMA-IR levels decreased and increased in those with and without baseline IR, respectively. (3) TG levels were independently associated with adiponectin levels in 12-month-old HCV core mice. (4) Comparable hepatic adiponectin expression was noted between tTA-HCV core DTM and tTA STM.

The comparisons of the baseline variables between the female and male CHC patients showed sex dimorphism in metabolic profiles [33] and adiponectin levels [16] and confirmed the reliability of the results of the current study. The factors consistently associated with adiponectin levels both pre-therapy in CHC patients and at 24 weeks post-therapy in SVR patients, such as sex and eGFR, disclosed their fundamental links of adiponectin, regardless of HCV infection. For example, higher adiponectin levels are usually noted in females than age-matched males [16], and circulating adiponectin level is elevated in chronic kidney disease patients [34]. By contrast, the pre-therapy only factors such as TG and FIB-4, suggested the potential links, direct or indirect, between HCV infection and adiponectin levels. Consistent with our previous studies based on CHC patients with interferon-based therapy [8, 17], both TG and hepatic fibrosis (FIB-4) were involved in HCV-associated adiponectin alteration. In particular, during HCV infection, adiponectin might affect HOMA-IR levels through TG, and the link was more evident among those with than those without baseline IR. Moreover, adiponectin levels consistently decreased after SVR, and HOMA-IR levels decreased and increased in those with and without baseline IR, respectively, in both interferon-treated [8] and DAA-treated CHC patients (Fig. 2). The HCV-associated factors for adiponectin might account for the decreasing trend of adiponectin levels, since low TG and high FIB-4 levels in CHC patients before viral clearance [8] lead to high baseline adiponectin levels; after viral clearance, reversal of hypotriglycemia and improved hepatic fibrosis [3, 8] cause decreased adiponectin levels. On the other hand, several pro-inflammatory cytokines such as tumor necrosis factor-α (TNF-α) and interleukin 6, are reported to down-regulate the expression of adiponectin, resulting in decreased serum adiponectin levels [18, 35]. In particular, activation of the TNF-α system has a pivotal role in the inflammatory process of CHC, and TNF-α levels correlate with the degree of inflammation [36]. Anyhow, we trust that the inflammation was improved in CHC patients after viral clearance, evidenced by the decreased ALT and NLR levels in SVR patients. Thus, although TNF-α levels were not surveyed in the current study, TNF-α is less likely to be the culprit for decreased adiponectin levels in SVR patients. Of note, the direct association between HOMA-IR and adiponectin levels in SVR patients following interferon-based therapy [8] vanished in SVR patients following DAA therapy (Fig. 2). The various metabolic or immune microenvironments following various therapeutic regimens might account for the discrepancies. For example, ferritin levels reflect the iron homeostasis [37] and are involved in a wide range of physiologic and pathologic processes [38]; the serum ferritin levels increased in SVR patients following interferon-based therapy [39] but decreased SVR patients following DAA therapy [40, 41]. Although the multivariate analyses in the current study did not show any independent role for ferritin levels in adiponectin and HOMA-IR levels, and the potential impact from ferritin on adiponectin and hOMA-IR levels, if any, might be indirect.

As the 12-month-old mice is equivalent to 58-year-old men [42], and the mean age of our human cohort is 61.3 years, the association of TG levels with adiponectin levels in 12-month-old HCV core transgenic mice echoes the crucial role of TG in affecting adiponectin levels in CHC patients and suggests the potential impact of HCV core on adiponectin levels. HCV core exhibits prominent topological relationship with intrahepatic lipid vesicles, which are mainly composed of TG [31]. Interestingly, the association between TG and adiponectin levels was not significant in the 2-month-old mice, which are equivalent to 20-year-old men [42]. Thus, the link between adiponectin and TG levels might not be evident until years of HCV infection in human. While that tTA-HCV core DTM (with hepatic steatosis) and tTA STM (without hepatic steatosis) had comparable hepatic adiponectin expression despite the different serum adiponectin levels [32] indicates that through interaction with TG, HCV core-related hepatic steatosis might alter adiponectin levels, via extrahepatic way.

## Conclusions

Taken together, TG and fibrosis-4 levels are associated with HCV-specific alteration of adiponectin levels. Adiponectin may affect insulin sensitivity through TG during HCV infection. In DAA-treated CHC patients, after viral clearance, adiponectin levels decreased and the link function of TG between adiponectin and insulin sensitivity vanished. Additionally, HCV core with subsequent hepatic steatosis might affect extrahepatic adiponectin expression through TG. These findings might pave the way to probe the therapeutic target for cardiometabolic events in CHC patients after SVR following DAA therapy.

## Methods

### Human study

The study group comprised subjects aged 18 years or older with CHC. Subjects with human immunodeficiency virus, hepatitis B infection, hemochromatosis, primary biliary cholangitis, primary sclerosing cholangitis, autoimmune hepatitis or malignancy and recipients of solid organ transplants were excluded. CHC was defined as detectable HCV RNA for > 24 weeks.

A total of 427 CHC patients were consecutively recruited at a tertiary referral center between May 2015 and December 2019. Of the 427 patients, 358 (Fig. 1) had completed a course of anti-HCV therapy with various DAA combination (Additional file 1: Table S1) according to the reimburse policy of Bureau of National Health Insurance of the country and had been followed ≥ 24 weeks after completion of DAA therapy. HCV RNA levels, genotypes, and single-nucleotide polymorphisms of interferon-λ3-rs12979860 were assessed as previously described [5, 7, 21–25]. Several baseline factors including sex, age, body mass index, HCV genotype, levels of HCV RNA, eGFR, UA, TC, TG, fasting glucose, fasting insulin, HOMA-IR [fasting insulin (μU/mL) × fasting glucose (mmol/L)/22.5], ALT and FIB-4 [(Age (years) × aspartate transaminase (U/L)/ (Platelets (10^9^/L) × (√(ALT (U/L))] index, MELD score, ferritin, adiponectin (R&D Systems, MN, USA) and the presence of hepatic cirrhosis were checked and recorded for all enrolled patients at baseline. Biochemical tests were performed at the clinical pathology laboratories of the hospital using routine automated techniques. An SVR was defined as undetectable levels of HCV RNA 12 weeks after the completion of therapy. In total, 353 had SVRs (Fig. 1). An IR was defined as HOMA-IR ≥ 2.5 [8]. The aforementioned variables were surveyed among the SVR patients at 24 weeks after the completion of therapy.

### Animal study

tTA-HCV core DTM in which expression of the HCV core gene was suppressible by tetracycline were raised as previously described [29–32]. These mice were raised and maintained in the specific-pathogen-free rooms of the animal center of the hospital, the experimental mice were acquired from the animal center of the hospital. For both the DTM and tTA STM, analyses of HCV core protein expression was performed by IHC staining as described previously [29–32], fat vesicles were identified by Oil Red O staining of frozen sections according to the manufacturer's protocol (BioGenex, Fremont, CA), and IHC studies of adiponectin (Novus Biologicals) were performed using frozen liver samples according to the manufacturer's protocols. Protein expression intensity was determined as described previously [10]. The fasting serum levels of adiponectin (R&D Systems, Minneapolis, MN) and insulin (Crystal Chem, Downers Grove, IL) were surveyed by using enzyme‐linked immunosorbent assay bioassay kits according to manufacturer's protocols in 2-month-old (males, n = 20, females, n = 20) and 12-month-old DTM (males, n = 16, females, n = 14) and in 2-month-old (males, n = 20, females, n = 20) and 12-month-old t-TA STM (males, n = 15, females, n = 15). The assays for fasting serum glucose, TG, TC and ALT levels (Vitros DT60 II Chemistry System; Johnson & Johnson, Rochester, NY) were adopted for using tail blood according to the manufacturer's protocol in 2-month-old (males, n = 20, females, n = 20) and 12-month-old DTM (males, n = 16, females, n = 14). After the end of the experiments, all the mice were sacrificed with CO2 euthanasia by using the home cages.

A schematic flow chart for the enrolled patients and transgenic mice was shown as Fig. 1.

### Statistics

All statistical analyses were performed using the Statistical Package for Social Science (SPSS package version 21, SPSS Inc., Chicago, IL, USA) or MedCalc (MedCalc ver. 12.4, MedCalc Software Corp., Acacialaan, Ostend, Belgium) software. The continuous variables are summarized as means ± standard deviations (SD), and the categorical variables are summarized as frequencies and percentages. The t-tests or Chi Square tests were applied for compared the variables between 2 groups while indicated. Multivariate linear regression models were used to assess the relationships between various dependent and independent factors by adjusting for all independent variables with *p* values < 0.1 in the univariate analyses. Repeated measures or Wilcoxon signed-rank tests were performed for the same variable measured in the same or matched subjects ≥ 2 time periods. Statistical significance was defined at the 5% level based on two-tailed tests.

## Supplementary Information

**Additional file 1**.** Supplementary Table 1**. Various DAA combinations used in the study.** Supplementary Table 2**. Associations of HOMA-IR levels in CHC patients with baseline IR at baseline.** Supplementary Table 3**. Associations of HOMA-IR levels in CHC patients without baseline IR at baseline.** Supplementary Table 4**. Associations of HOMA-IR levels in SVR patients with baseline IR at 24 weeks post-therapy.** Supplementary Table 5**. Associations of HOMA-IR levels in SVR patients without baseline IR at 24 weeks post-therapy

## Data Availability

The datasets used and/or analyzed during the current study are available from the corresponding author on reasonable request. Because part of our data will be further analyzed for other study, we thus did not deposit the datasets in any publicly available repositories.

## Abbreviations

HCV: Hepatitis C virus
SVRs: Sustained virological responses
DAA: Direct-acting antivirals
eGFR: Estimated glomerular filtration rate
HOMA-IR: Homeostatic model assessment for insulin resistance
HCC: Hepatocellular carcinoma
IR: Insulin resistance
CHC: Chronic HCV infection
IHC: Immunohistochemistry
BMI: Body mass index
TG: Triglycerides
tTA: Tetracycline transactivator
DTM: Double transgenic mice
STM: Single transgenic mice
