# Supplementary material for: Critical role of triglycerides for adiponectin levels in hepatitis C: a joint study of human and HCV core transgenic mice
Source: BMC Immunol. 2021 Aug 11;22:54. doi: 10.1186/s12865-021-00445-5 (PMC8359585; doi:10.1186/s12865-021-00445-5)
Supplement: Supplementary file 1 — Additional file 1. Supplementary Table 1. Various DAA combinations used in the study. Supplementary Table 2. Associations of HOMA-IR levels in CHC patients with baseline IR at baseline. Supplementary Table 3. Associations of HOMA-IR levels in CHC patients without baseline IR at baseline. Supplementary Table 4. Associations of HOMA-IR levels in SVR patients with baseline IR at 24 weeks post-therapy. Supplementary Table 5. Associations of HOMA-IR levels in SVR patients without baseline IR at 24 weeks post-therapy [file 12865_2021_445_MOESM1_ESM.doc]

**Supplementary Table 1. Various DAA combinations used** in the study.

| Genotype 1 |  | Genotype 2 |  | Genotype 3 |  | Genotype 4 |  | Genotype 5 |  | Genotype 6 |  |
| --- | --- | --- | --- | --- | --- | --- | --- | --- | --- | --- | --- |
| DAA | D(W) | DAA | D(W) | DAA | D(W) | DAA | D(W) | DAA | D(W) | DAA | D(W) |
| Asunaprevir (100 mg, bid) with Daclatasvir (60 mg, qd) | 24 | Sovaldi (Sofosbuvir 400 mg qd) with Ribavirin | 12 |  |  |  |  |  |  |  |  |
| Viekirax (Ombitasvir 12.5mg/Paritaprevir 75mg/Ritonavir 50 mg qd) with Exviera (250 mg bid) | 12-24 |  |  |  |  |  |  |  |  |  |  |
| Zepatier (Elbasvir 50 mg/Grazoprevir 100 mg, qd) | 12-16 |  |  |  |  |  |  |  |  |  |  |
| Harvoni (Ledipasvir 90 mg /Sofosbuvir 400 mg qd) with/without Ribavinin (800-1400 mg/day, in two divided doses | 12 | Harvoni (Ledipasvir 90 mg /Sofosbuvir 400 mg qd) with/without Ribavinin (800-1400 mg/day, in two divided doses | 12 |  |  | Harvoni (Ledipasvir 90 mg /Sofosbuvir 400 mg qd) with/without Ribavinin (800-1400 mg/day, in two divided | 12 | Harvoni (Ledipasvir 90 mg /Sofosbuvir 400 mg qd) with/without Ribavinin (800-1400 mg/day, in two divided | 12 | Harvoni (Ledipasvir 90 mg /Sofosbuvir 400 mg qd) with/without Ribavinin (800-1400 mg/day, in two divided | 12 |
| Mavyret (glecaprevir 100mg/pibrentasvir 40mg, 3# qd) | 8-16 | Mavyret (glecaprevir 100mg/pibrentasvir 40mg, 3# qd) | 8-12 | Mavyret (glecaprevir 100mg/pibrentasvir 40mg, 3# qd) | 8-16 | Mavyret (glecaprevir 100mg/pibrentasvir 40mg, 3# qd) | 8-12 | Mavyret (glecaprevir 100mg/pibrentasvir 40mg, 3# qd) | 8-12 | Mavyret (glecaprevir 100mg/pibrentasvir 40mg, 3# qd) | 8-12 |
| Epclusa (sofosbuvir 400 mg/velpatasvir 100 mg qd) with or without Ribavirin | 12 | Epclusa (sofosbuvir 400 mg/velpatasvir 100 mg qd) with or without Ribavirin | 12 | Epclusa (sofosbuvir 400 mg/velpatasvir 100 mg qd) with or without Ribavirin | 12 | Epclusa (sofosbuvir 400 mg/velpatasvir 100 mg qd) with or without Ribavirin | 12 | Epclusa (sofosbuvir 400 mg/velpatasvir 100 mg qd) with or without Ribavirin | 12 | Epclusa (sofosbuvir 400 mg/velpatasvir 100 mg qd) with or without Ribavirin | 12 |

DAA: direct-acting antiviral agent; D: duration: W: weeks.

Supplementary Table 2. Associations of HOMA-IR levels in CHC patients with baseline IR at baseline

| Baseline factors | Univariate analyses | | Multivariate analyses | |
| --- | --- | --- | --- | --- |
|  | 95% CI of β (β) | *p* values | 95% CI of β (β) | *p* values |
| Male, yes | -7.042~4.516 (-1.263) | 0.667 |  |  |
| Age (years) | -0.244 ~0.198 (-0.023) | 0.836 |  |  |
| BMI (kg/m^2^) | -0.809~0.676 (-0.066) | 0.86 |  |  |
| HCV genotype | 7.86~18.6 (13.3) | <0.001 | 7.606~18.443 (13.024) | 0.001 |
| Log HCV RNA (logIU/mL) | -0.145~2.271 (1.063) | 0.084 | -0.263 ~2.4 (1.069) | 0.433 |
| ALT (U/L) | -0.034~0.018 (-0.008) | 0.531 |  |  |
| eGFR (mL/min/1.73 m^2^) | -0.074~0.06 (-0.007) | 0.843 |  |  |
| TG (mg/dL) | -0.009~0.074 (0.032) | 0.127 |  |  |
| TC (mg/dL) | -0.008~0.158 (0.075) | 0.076 | -0.045 ~0.019 (-0.013) | 0.932 |
| Adiponectin (μg/mL) | -0.67~0.226 (-0.222) | 0.33 |  |  |
| Uric acid (mg/dL) | -0.292 ~2.39 (0.149) | 0.896 |  |  |
| NLR | -2.63 ~2.659 (0.013) | 0.992 |  |  |
| Platelet (10^3^/uL) | -8.76~4.579 (-2.092) | 0.537 |  |  |
| Liver cirrhosis, yes | -0.692~1.595 (0.451) | 0.438 |  |  |
| Fibrosis-4 score | -1.243 ~0.553 (-0.345) | 0.449 |  |  |
| IFNL3-rs12979860  CC genotype, yes | -3.578~3.872 (0.147) | 0.938 |  |  |

HOMA-IR: homeostatic model assessment for insulin resistance; CHC: chronic hepatitis C virus infection; IR: insulin resistance; CI: confidence interval; BMI: body mass index; HCV: hepatitis C virus; RNA: ribonucleic acid; ALT: alanine transaminase; eGFR: estimated glomerular filtration rate; TG: triglycerides; TC: total cholesterol; NLR: neutrophil lymphocyte ratio; IFNL3; interferon-λ3.

Supplementary Table 3. Associations of HOMA-IR levels in CHC patients without baseline IR at baseline

| Baseline factors | Univariate analyses | | Multivariate analyses | |
| --- | --- | --- | --- | --- |
|  | 95% CI of β (β) | *p* values | 95% CI of β (β) | *p* values |
| Male, yes | -0.061~0.127 (0.033) | 0.492 |  |  |
| Age (years) | 0.003~0.009 (0.006) | <0.001 | -0.007~0.007 (0.000) | 0.094 |
| BMI (kg/m^2^) | 0.042~0.072 (0.057) | <0.001 | 0.016~0.066 (0.041) | 0.002 |
| HCV genotype | -0.235~0.328 (0.047) | 0.745 |  |  |
| Log HCV RNA (logIU/mL) | -0.025~0.129 (0.052) | 0.184 |  |  |
| ALT (U/L) | 0.002~0.004 (0.003) | <0.001 | 0.000~0.002 (0.001) | 0.143 |
| eGFR (mL/min/1.73 m^2^) | -0.002~0.001 (0.000) | 0.575 |  |  |
| TG (mg/dL) | 0.001~0.002 (0.001) | <0.001 | -0.002~0.003 (0.000) | 0.777 |
| TC (mg/dL) | -0.002~0.000 (-0.001) | 0.086 | -0.001~0.004 (0.002) | 0.249 |
| Adiponectin (μg/mL) | -0.052 ~0.000 (-0.026) | 0.053 | -0.05 ~0.01 (-0.02) | 0.196 |
| Uric acid (mg/dL) | 0.04~0.105 (0.073) | <0.001 | -0.24 ~0.079 (0.027) | 0.301 |
| NLR | -0.038 ~0.051 (0.006) | 0.78 |  |  |
| Platelet (10^3^/uL) | -0.002~-0.001 (-0.002) | <0.001 | -0.002 ~0.000 (-0.001) | 0.121 |
| Liver cirrhosis, yes | 0.266~0.566 (0.396) | <0.001 | -0.159 ~0.297 (0.071) | 0.538 |
| Fibrosis-4 score | -0.03 ~0.112 (0.041) | 0.251 |  |  |
| IFNL3-rs12979860  CC genotype, yes | -0.163~0.306 (0.071) | 0.547 |  |  |

HOMA-IR: homeostatic model assessment for insulin resistance; CHC: chronic hepatitis C virus infection; IR: insulin resistance; CI: confidence interval; BMI: body mass index; HCV: hepatitis C virus; RNA: ribonucleic acid; ALT: alanine transaminase; eGFR: estimated glomerular filtration rate; TG: triglycerides; TC: total cholesterol; NLR: neutrophil lymphocyte ratio; IFNL3; interferon-λ3.

Supplementary Table 4. Associations of HOMA-IR levels in SVR patients with baseline IR at 24 weeks post-therapy

| 24-week post-therapy factors | Univariate analyses | | Multivariate analyses | |
| --- | --- | --- | --- | --- |
|  | 95% CI of β (β) | *p* values | 95% CI of β (β) | *p* values |
| Male, yes | -0.166~0.778 (0.306) | 0.203 |  |  |
| Age, (years) | -0.079 ~0.036 (-0.022) | 0.456 |  |  |
| BMI (kg/m^2^) | 0.061~0.418 (0.239) | 0.009 | 0.011~0.429 (0.22) | 0.039 |
| ALT (U/L) | 0.018~0.098 (0.056) | 0.008 | -0.003~0.096 (0.046) | 0.066 |
| eGFR (mL/min/1.73 m^2^) | -0.0011~0.042 (0.016) | 0.238 |  |  |
| TG (mg/dL) | 0.000~0.014 (0.007) | 0.043 | -0.003~0.012 (0.004) | 0.273 |
| TC (mg/dL) | -0.031~0.011 (-0.01) | 0.35 |  |  |
| Adiponectin (μg/mL) | -0.404 ~0.211 (-0.096) | 0.536 |  |  |
| Uric acid (mg/dL) | -0.332~0.698 (0.181) | 0.481 |  |  |
| NLR | -0.724~0.851 (0.063) | 0.873 |  |  |
| Platelet (10^3^/uL) | -0.026~-0.001 (-0.013) | 0.04 | -0.028~0.014 (-0.007) | 0.494 |
| Liver cirrhosis, yes | -0.907~2.296 (0.695) | 0.392 | -0.482~0.752 (0.135) | 0.135 |
| Fibrosis-4 score | -0.051~0.679 (0.317) | 0.09 |  |  |
| IFNL3-rs12979860  CC genotype, yes | -3.72~3.62 (-1.682) | 0.106 |  |  |

HOMA-IR: homeostatic model assessment for insulin resistance; CHC: chronic hepatitis C virus infection; IR: insulin resistance; CI: confidence interval; BMI: body mass index; HCV: hepatitis C virus; RNA: ribonucleic acid; ALT: alanine transaminase; eGFR: estimated glomerular filtration rate; TG: triglycerides; TC: total cholesterol; NLR: neutrophil lymphocyte ratio; IFNL3; interferon-λ3.

**Supplementary Table 5**. Associations of HOMA-IR levels in SVR patients without baseline IR at 24 weeks post-therapy

| 24-week post-therapy factors | Univariate analyses | | Multivariate analyses | |
| --- | --- | --- | --- | --- |
|  | 95% CI of β (β) | *p* values | 95% CI of β (β) | *p* values |
| Male, yes | -1.221~1.742 (0.26) | 0.729 |  |  |
| Age, (years) | -0.014 ~0.023 (0.004) | 0.639 |  |  |
| BMI (kg/m^2^) | 0.06~ 0.202 (0.131) | <0.001 | 0.001~0.148 (0.201) | 0.047 |
| ALT (U/L) | 0.04~0.073 (0.056) | <0.001 | 0.014~0.057 (0.309) | 0.002 |
| eGFR (mL/min/1.73 m^2^) | -0.006~0.007 (0.001) | 0.875 |  |  |
| TG (mg/dL) | 0.007~0.015 (0.011) | <0.001 | -0.001~0.01 (0.005) | 0.089 |
| TC (mg/dL) | -0.007~0.005 (-0.001) | 0.766 |  |  |
| Adiponectin (μg/mL) | -0.041 ~0.143 (0.051) | 0.276 |  |  |
| Uric acid (mg/dL) | -0.066~0.202 (0.068) | 0.317 |  |  |
| NLR | 0.099~0.46 (0.279) | 0.003 | 0.073~0.395 (0.272) | 0.005 |
| Platelet (10^3^/uL) | -0.013~0.00 (-0.007) | 0.059 | -0.005~0.002 (-0.001) | 0.382 |
| Liver cirrhosis, yes | -0.012~1.148 (0.568) | 0.055 | -0.188~0.869 (0.341) | 0.203 |
| Fibrosis-4 score | -0.069~0.263 (0.097) | 0.252 |  |  |
| IFNL3-rs12979860  CC genotype, yes | -0.49~1.005 (0.258) | 0.496 |  |  |

HOMA-IR: homeostatic model assessment for insulin resistance; CHC: chronic hepatitis C virus infection; IR: insulin resistance; CI: confidence interval; BMI: body mass index; HCV: hepatitis C virus; RNA: ribonucleic acid; ALT: alanine transaminase; eGFR: estimated glomerular filtration rate; TG: triglycerides; TC: total cholesterol; NLR: neutrophil lymphocyte ratio; IFNL3; interferon-λ3.
